# Establishing a glutamine metabolism-based model for predicting the prognosis of low-grade glioma

**DOI:** 10.3389/fgene.2022.1030837

**Published:** 2022-11-22

**Authors:** Mingshi Zhang, Mingjun Li, Jinrui Liu, Zhicheng Gu, Yanmei Lu, Yu Long, Yuyi Hou

**Affiliations:** ^1^ Department of Neurosurgery, The First Affiliated Hospital of Jiamusi University, Jiamusi, China; ^2^ Department of Stomatology, The First Affiliated Hospital of Jiamusi University, Jiamusi, China

**Keywords:** glutamine, molecular typing, low-grade glioma, prognostic model, decision tree

## Abstract

**Background:** The natural history of patients with low-grade glioma (LGG) varies widely, but most patients eventually deteriorate, leading to poor prognostic outcomes. We aim to develop biological models that can accurately predict the outcome of LGG prognosis.

**Methods:** Prognostic genes for glutamine metabolism were searched by univariate Cox regression, and molecular typing was constructed. Functional enrichment analysis was done to evaluate potential prognostic-related pathways by analyzing differential genes in different subtypes. Enrichment scores of specific gene sets in different subtypes were measured by gene set enrichment analysis. Different immune infiltration levels among subtypes were calculated using algorithms such as CIBERSORT and ESTIMATE. Gene expression levels of prognostic-related gene signatures of glutamine metabolism phenotypes were used to construct a RiskScore model. Receiver operating characteristic curve, decision curve and calibration curve analyses were used to evaluate the reliability and validity of the risk model. The decision tree model was used to determine the best predictor variable ultimately.

**Results:** We found that C1 had the worst prognosis and the highest level of immune infiltration, among which the highest macrophage infiltration can be found in the M2 stage. Moreover, most of the pathways associated with tumor development, such as MYC_TARGETS_V1 and EPITHELIAL_MESENCHYMAL_TRANSITION, were significantly enriched in C1. The wild-type IDH and MGMT hypermethylation were the most abundant in C1. A five-gene risk model related to glutamine metabolism phenotype was established with good performance in both training and validation datasets. The final decision tree demonstrated the RiskScore model as the most significant predictor of prognostic outcomes in individuals with LGG.

**Conclusion:** The RiskScore model related to glutamine metabolism can be an exceedingly accurate predictor for LGG patients, providing valuable suggestions for personalized treatment.

## Introduction

Low-grade glioma (LGG) is a rare group of primary central nervous system tumors categorized by WHO as grades I and II, including diffuse astrocytomas and oligodendrogliomas (Louis et al., 2016). Usually, in an inactive state, while many tumors eventually evolve into fatal high-grade gliomas (Sanai et al., 2011). Due to the long asymptomatic natural history of these tumors, there is no certainty whether to give aggressive or delayed treatment. In addition, the timing of chemotherapy and radiotherapy after surgery to those individuals with few symptoms and limited lesions is not specified (Shaw et al., 2008; van den Bent et al., 2005). Most individuals with LGG express mutated isocitrate dehydrogenase (IDH) 1 or 2, which produce 2-hydroxyglutaric acid (2-HG), inducing glioma development and immunosuppressive effects in the tumor microenvironment (Kohanbash et al., 2017; Bunse et al., 2018).

The most prevalent amino acid in the human body, glutamine, is a precursor with numerous uses that contributes to several metabolic and biosynthetic processes (Altman et al., 2016). In 1955, cancer cells were shown to obtain glutamine from the local microenvironment to promote tumor growth (Eagle, 1955; Jin et al., 2016). Glutamine is not considered among the classically essential amino acids since glutamine synthase can synthesize it from glutamate and ammonia, certain tumors break down proteins by means of autophagy to release amino acids such as glutamine (Seo et al., 2016). Gamma (γ) (amide) nitrogen from glutamine is added to the synthesis of ribonucleic acid and hexosamine in the cytoplasm, producing glutamate. By generation of glutathione (GSH), cytoplasmic glutamate is essential for redox homeostasis and preventing oxidative stress in cells (Conrad and Sato, 2012). In addition to glutamine being an oncogene-dependent addiction for many cancer cells, it also promotes proliferative signaling. For instance, the glutamine influx molecule through SLC1A5 is closely associated with the efflux molecule through the SLC7A5/LAT1 transport protein (Dolgodilina et al., 2016). The SLC7A5/LAT1 transfer protein also allows leucine to enter cells and induces MTORC1-mediated cell growth. Moreover, the Warburg effect is triggered by the signal transduction molecules Akt, Ras, and AMPK to activate glycolytic enzymes, which causes the production of lactate, forcing cancer cells to switch to glutamine metabolism and satisfy the heightened energy needs. Through the activation of the glutaminase (GLS) and SLC1A5 genes during transcription, the proto-oncogene c-Myc increases glutamine catabolism (Hensley et al., 2013; Kim and Kim, 2013; Chen and Cui, 2015; Jin et al., 2016). Moreover, glutamine may be considered a conditionally essential amino acid for lymphocytes and numerous tumors because these cells require environmental absorption to survive because they consume more glutamine than they can produce (Lacey and Wilmore, 1990; Cluntun et al., 2017). Glutamine is also a key immunomodulator in the initiation and development of T-cell-mediated immunity (Pacheco et al., 2007). Tumors show characteristics related to elevated glutamine metabolism possibly limiting glutamine utilization by the immune system, resulting in a low overall survival of patients. Therefore, understanding the potential relationship between glutamine metabolism and cancer progression is a fundamental goal of cancer research.

In this study, prognostic genes of the glutamine metabolic pathway were used to identify stable molecular subtypes by consistent clustering and further compared clinical features, pathway and immune characteristics among subtypes. Finally, we identified glutamine metabolism phenotype-related genes by expression difference analysis and least absolute shrinkage and selection operator (LASSO) regression analysis. Furthermore, a risk model and a clinical prognostic model were constructed, to assist in the personalized treatment of individuals with LGG.

## Methods

### Data collection and processing

The analysis of this research was supported by the Sangerbox platform (Shen et al., 2022). We obtained RNA-Seq data of TCGA-LGG using The Cancer Genome Atlas (TCGA) and performed the preprocessing, including removing samples without clinical data and converting Ensembl to Gene symbol. The average of the expression values was achieved when multiple identical Gene Symbols existed. After preprocessing, 506 samples were remained. In addition, we downloaded "mRNAseq_693 (batch 1)" and " mRNAseq_325 (batch 2)" datasets from Chinese Glioma Genome Atlas (CGGA) database (http://www.cgga.org.cn/). The samples with histological type of Glioblastoma (GBM) were excluded. ComBat” function in the Sva R package was conducted to remove the batch effects of "mRNAseq_693 (batch 1)" and " mRNAseq_325 (batch 2)" (named as CGGA dataset), and 408 samples were finally included.

### Source of glutamine metabolism-related genes

Genes related to glutamine metabolism were obtained from the “GOBP_GLUTAMINE_FAMILY_AMINO_ACID_METABOLIC_PROCESS” in Molecular Signatures Database (MSigDB) (https://www.gsea-msigdb.org/gsea/msigdb/human/geneset/GOBP_GLUTAMINE_FAMILY_AMINO_ACID_METABOLIC_PROCESS, Supplementary Table S1) (Liberzon et al., 2015).

### Identification of molecular subtypes of glutamine metabolism-related genes

The consensus matrix was constructed by ConsensusClusterPlus, and cluster typing of the processed TCGA samples was done (Wilkerson and Hayes, 2010). The expression data of glutamine metabolism-related genes were used to obtain the molecular subtypes of the samples. We did 500 bootstraps using the "km" algorithm and "1—Pearson correlation" as the metric distance, with each bootstrap having 80% of the individuals in the training set. The number of clusters was set from 2 to 10, and the molecular subtypes of the samples were obtained by measuring the consistency matrix and consistency cumulative distribution function. In the TCGA dataset, we also explored the genomic alterations in these three molecular subtypes. In this study, we obtained data on the molecular properties of the TCGA dataset from the previous pan-cancer studies (Thorsson et al., 2018).

### Construction of risk model

The identified molecular subtypes recognized differentially expressed genes (DEGs) among subtypes, and then DEGs (|log2FC|>1 & *p* < 0.01) were selected. Finally, the following equation was used to determine the risk scores for individual patients: RiskScore = Σ *β*i × Expi, Expi refers to the gene expression level of the prognostic-related gene signature of the glutamine metabolism phenotype, and *β* is the Cox regression coefficient of the relevant gene. The z-score was then performed, and individuals were sorted into high- and low-risk groups keeping the threshold at "0", and for prognostic analysis, we plotted survival curves following the Kaplan-Meier method. The significance of variations was determined by the log-rank test.

### Gene set enrichment analysis (GSEA)

Gene set enrichment analysis (GSEA) was done and all candidate gene sets from the Hallmark database were utilized to assess the pathways of various biological activities in various molecular subtypes (Liberzon et al., 2015). Both inflammatory signature-related gene sets and angiogenesis-related gene sets were obtained from literature reports (Masiero et al., 2013; Liu et al., 2020). Considering that interferon (IFN)-γ is a cytokine essential in immunomodulation and anti-cancer immunity, we downloaded the GOBP_RESPONSE_TO_INTERFERON_GAMMA gene set from the Gene Ontology (GO) database. Single sample Gene set enrichment analysis (ssGSEA) was used to calculate the enrichment fraction of a specific gene set.

### Calculation of tumor microenvironment cell invasion abundance

We determined the relative abundance of 22 types of immune cells in LGG using the CIBERSORT method (https://cibersort.stanford.edu/). We also used ESTIMATE software to measure the proportion of immune cells (Yoshihara et al., 2013). T-cell inflammatory gene expression profile (GEP), programmed death ligand 1 (PD-L1) expression, and tumor mutational burden (TMB) are three biomarkers whose responses to anti-programmed cell death 1 (PD-1) treatment may be predicted by the T-Cell-Inflamed Gene-Expression Profile score (Ott et al., 2019). Cytolytic activity score (CYT) was used to report the level of cytotoxic T cell activation (Takahashi et al., 2020).

### Correlation analysis of risk score and drug sensitivity

We used the R package "pRRophetic" for drug IC50 prediction (Geeleher et al., 2014). Drug response prediction was performed against the expression matrix.

### Differential gene acquisition between subtypes and GO/KEGG functional enrichment analysis

Genes with differential expression between C1, C2, and C3 vs. others in the TCGA-LGG cohort were computed using the R package "limma" (Ritchie et al., 2015). The R package "clusterProfiler" conducted a functional enrichment analysis (Yu et al., 2012). Species were set to *Homo sapiens*, and the entries analyzed contained all GO and Kyoto Encyclopedia of Genes and Genomes (KEGG) entries with the *p*-value adjustment method False Discovery Rate (FDR).

### Protein interaction network and key protein module

We created a protein-protein interaction (PPI) network with the help of STRING online tool (https://string-db.org/) and Cytoscape 3.9.1 to study essential proteins of differential genes in the subtypes. We used the MCODE plug-in in Cytoscape in this network to discover network modules.

### Prognostic gene correlation analysis

With the help of univariate COX regression, prognostically significant genes were identified. Further, using the R package "glmnet" (Friedman et al., 2010), LASSO regression was conducted to lower the number of genes in order to obtain prognostically significant genes linked to the glutamine metabolism phenotype. Additionally, DEGs were further compressed to lower the genes’ number for the risk model (Friedman et al., 2010). Stepwise multi-factor regression analysis was then performed utilizing the Akaike Information Criterion (AIC) Information Criterion, which considers the model’s statistical fit and the number of parameters that were appropriate for it. The stepAIC strategy in R package "MASS" starts with the most complicated model and sequentially removes each variable to lower the AIC (Zhang, 2016). A smaller value indicated better performance of the model, which indicates that the model obtained an eligible fitting degree with less number of parameters. The R package "timeROC" was employed to plot the receiver operating characteristic (ROC) to determine the model’s strength (Heagerty et al., 2000). Decision trees were constructed for different variables to determine the best indicator. Calibration curve and decision curve analysis (DCA) were utilized to assess the model’s predictive reliability and accuracy.

### Statistical analysis

The R platform was employed to conduct all statistical analyses. Log-rank test was done in both Cox regression and Kaplan-Meier survival analyses. Kruskal–Wallis test was employed to determine the variation among the three groups, and for determining the difference between the two groups, the Wilcoxon test was done. ANOVA was conducted to evaluate the distribution of the clinicopathological feature in different subtypes (ns, *p* ≥ 0.05; *p < 0.05; **p < 0.01; ***p < 0.001).

## Results

### Molecular typing based on genes linked with glutamine metabolism

**FIGURE 1:**
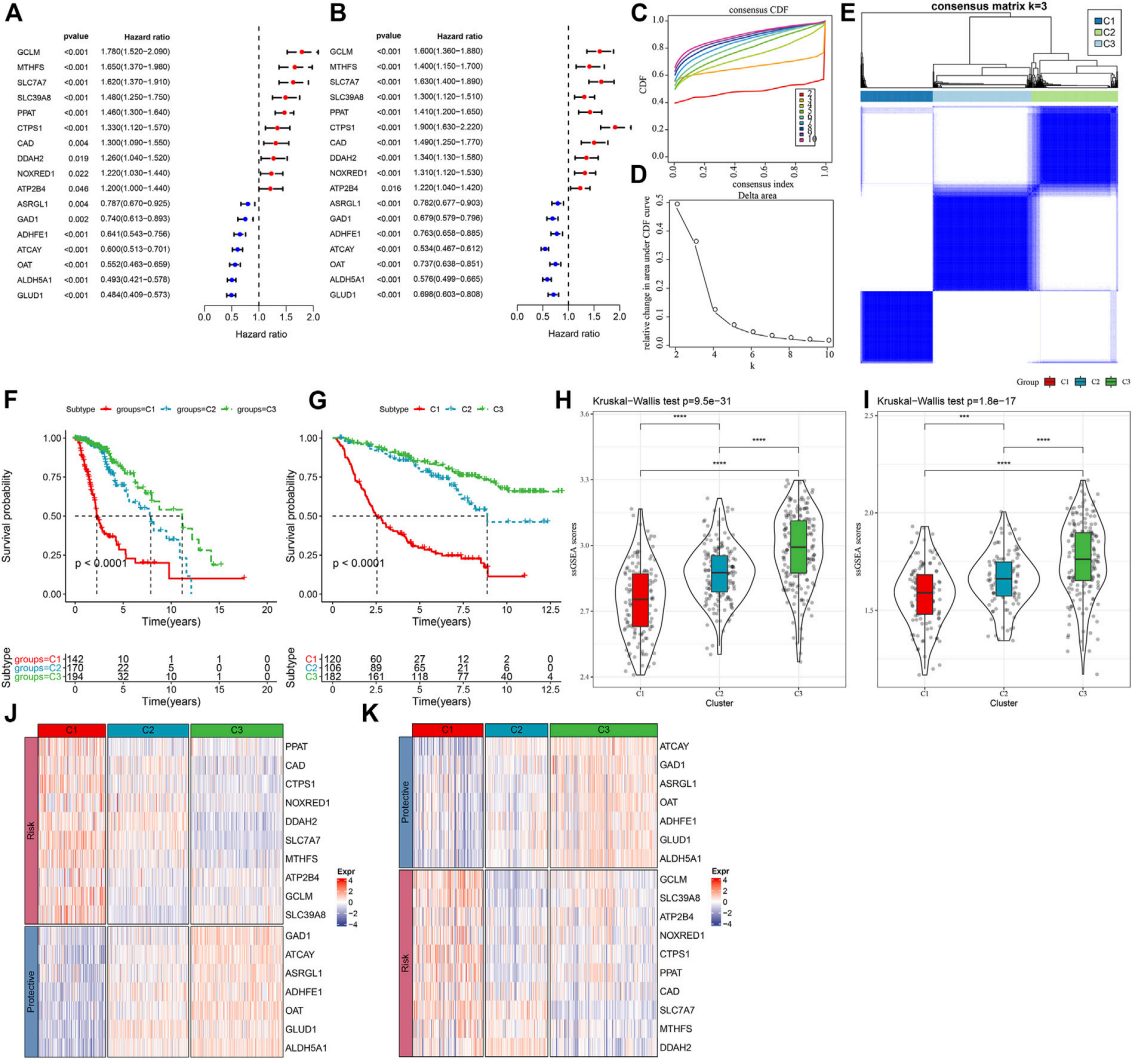
Molecular typing in accordance with the glutamine metabolism genes **(A)** Forest plot of glutamine metabolism crossover genes in TCGA dataset; **(B)** Forest plot of glutamine metabolism crossover genes in CGGA cohort. **(C)** CDF curve of TCGA dataset samples. **(D)** CDF Delta area curve of TCGA dataset samples. Delta area curve of consensus clustering, indicating the relative change in area under the CDF curve for each category number k in comparison with k – 1. The horizontal axis is for the category number k, and the vertical axis is for the relative change in area under the CDF curve. **(E)** Heat map of sample clustering at consensus k = 3. **(F)** KM curves of the relationship between the prognosis of the three subtypes in the TCGA dataset. **(G)** KM curves of the relationship between the prognosis of the three subtypes in the CGGA dataset. **(H)** Differences in glutamine metabolism scores between different molecular subtypes in the TCGA-LGG cohort. **(I)** Differences in glutamine metabolism scores in different molecular subtypes in the CGGA dataset. **(J)** Heat map of expression of prognostically significant glutamine metabolism-related genes in different subtypes in the TCGA dataset. **(K)** Heat map of expression of prognostically significant glutamine metabolism-related genes in different subtypes in the CGGA dataset.

In order to assess the expression patterns of genes linked with glutamine metabolism, a univariate Cox regression analysis was done using LGG samples from the TCGA-LGG and CGGA datasets containing clinical information. The results showed that 36 glutamine metabolism genes were prognostically associated with LGG in the TCGA-LGG dataset (*p* < 0.05) and 32 glutamine metabolism genes with significant prognoses in the CGGA dataset. Further, we selected glutamine metabolism genes with significant prognosis in both TCGA and CGGA, and 17 glutamine metabolism genes were selected (Figures 1A, B). Subsequently, consistent clustering was utilized to sort the TCGA data set in accordance with the 17 prognostically significant glutamine metabolism gene expression data, determined the optimal number of clusters based on the cumulative distribution function (CDF), and observed the CDF Delta area curve from which we could see that the Cluster selection of three had more stable clustering results (Figures 1C, D). At the end, k = 3 was chosen to get three molecular subtypes (Figure 1E).

Moreover, to assess the prognostic properties of these three molecular subtypes, considerable prognostic variations among them were noted (Figure 1F). Overall, C3 had an improved prognosis, while a worse prognosis was observed in the C1 subtype. Additionally, we classified patients in the CGGA dataset and finally identified three subtypes, and the prognostic outcomes differed significantly among the subtypes (Figure 1G). In addition, we also calculated the ssGSEA scores of glutamate metabolism for every individual with LGG in the TCGA dataset. A high glutamate metabolism score was found in the C1 subtype and C3 had the lowest glutamate metabolism score (Figure 1H). Similar phenomenon was observed in the CGGA cohort (Figure 1I, Supplementary Table S2). We also compared the differential expression of 17 glutamine metabolism genes in the distinct molecular subtypes that we defined and found that in two independent datasets, enhanced expression of the overall Risk genes was seen in the C1 subtype. In contrast, protective genes were expressed increasingly in the C3 subtype (Figures 1J,K).

### Clinicopathological features among molecular subtypes

**FIGURE 2:**
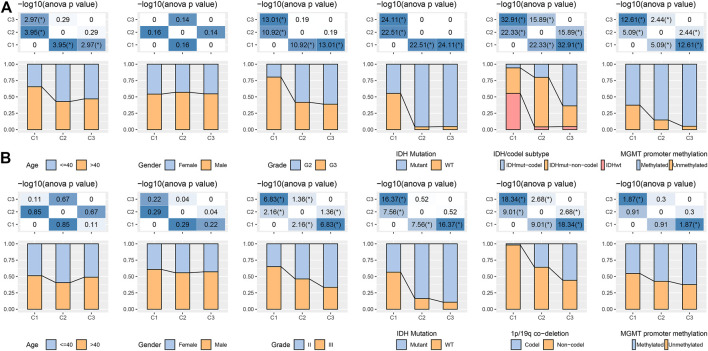
Distribution characteristics of different molecular subtypes in each clinical variable **(A)** Clinicopathological characteristics of molecular subtypes in the TCGA dataset. **(B)** Clinicopathological characteristics of molecular subtypes in the CGGA cohort; therein, the lower half shows the proportion, and the upper half shows the statistical significance of the difference in distribution between the two -log10 (*p*-value).

In the TCGA and CGGA datasets, a comparison was made regarding the distribution of various clinical properties in the three molecular subtypes to find the difference in clinical properties among them (Figure 2). No major variation was observed in the gender among the three subtypes, while in terms of grade, patients with the C1 subtype were more likely to be Grade 3 (G3) and more likely to be G2 in C2 and C3 subtypes. We discovered that the frequency of IDH mutations was much higher in the C1 subtype, which had a poor prognosis in comparison with the other two subtypes. Additionally, different IDH mutation types more reduced in the C1 subtype. In terms of 1p19q association deletion, the C3 subtype had significantly higher 1p19q association deletion than the C1 and C2 molecular subtypes.

Regarding MGMT promoter methylation, C2 and C3 subtypes had significantly higher MGMT promoter methylation than C1 subtypes (Figure 2A). We also compared the differences in age, sex, Grade, IDH mutation, 1p19q association deletion, and MGMT promoter methylation in CGGA. We found that age and gender were also not significantly different in CGGA. IDH mutation and 1p19q association deletion was significantly higher in C2 and C3 than in C1, these outcomes are similar to the phenomenon observed in TCGA (Figure 2B).

### Genomic landscape among molecular subtypes

**FIGURE 3:**
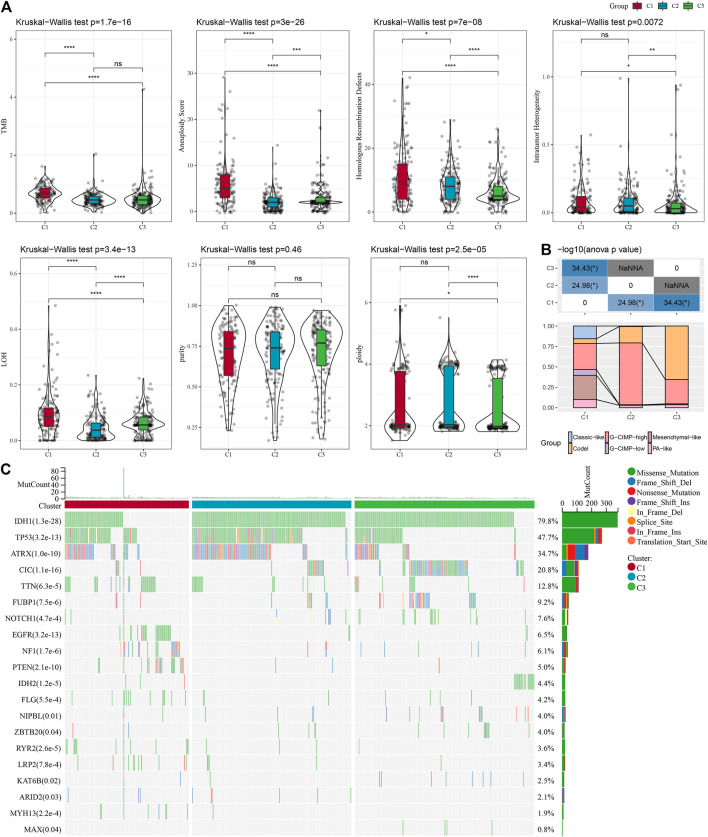
Mutation load in different molecular subtypes: genomic alterations in molecular subtypes of TCGA cohort. **(A)** Comparison of Tumor mutation burden, Aneuploidy Score, Homologous Recombination Defects, Intratumor Heterogeneity,LOH,purity, ploidy. **(B)** Comparison of the three molecular subtypes with immune molecular subtypes. **(C)** Somatic mutations in the three molecular subtypes (chi-square test). *p < 0.05; **p < 0.01; ***p < 0.001; and ****p < 0.0001.

It can be seen that C1 subtypes show higher TMB, aneuploidy score, homologous recombination defects, intratumor heterogeneity, and loss of heterozygosity (LOH) (Figure 3A). Moreover, extra molecular subtypes were also given in this study, and we also compared these six molecular subtypes with our three molecular subtypes and found more "Codel" molecular subtypes in the C3 and more "G-CIMP-high" molecular subtypes in the C2 (Figure 3B). In addition, a comparison of the variations in the mutations among different molecular subtypes was made, and the outcomes revealed that the top 20 genes with significant differences, from which we could see that the mutation frequencies of IDH1, TP53, and other genes were significantly different between the three molecular subtypes (Figure 3C).

### Pathway characteristics among various molecular subtypes

**FIGURE 4:**
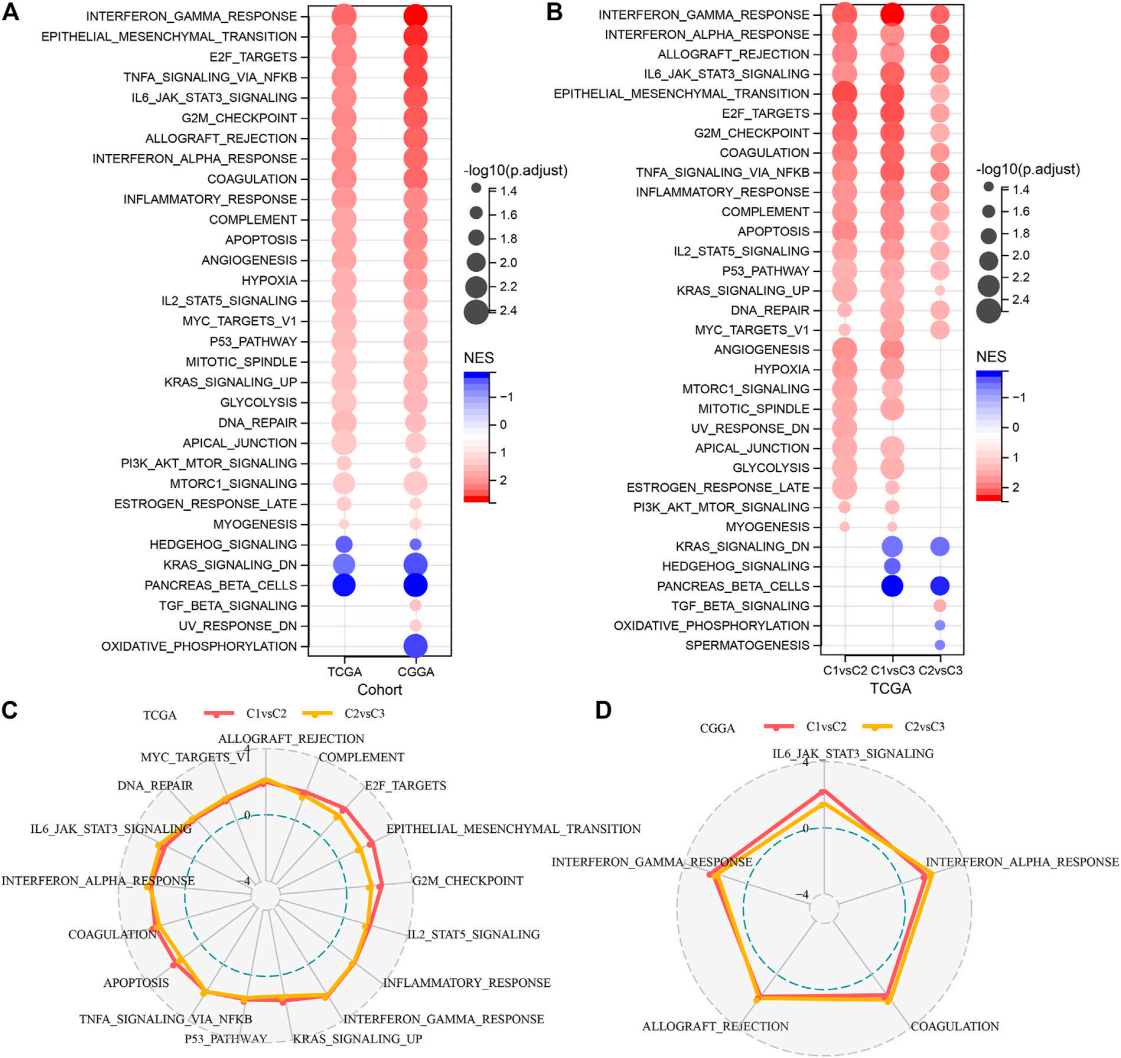
Significantly activated pathways in various molecular subtypes **(A)** Bubble chart of GSEA results for C1 vs. C3 subtypes in two LGG cohorts. **(B)** Bubble chart of GSEA results for various molecular subtypes compared in the TCGA-LGG cohort. **(C)** Radar chart of C1 vs. C2, C2 vs. C3 consistently activated pathways in the TCGA-LGG dataset. **(D)** Radar chart of C1vsC2, and C2vsC3 consistent activation pathways in the CGGA dataset.

It is observed that the TCGA dataset is significantly enriched to 29 pathways in the C1 subtype, and overall, the activated pathways mainly contain some pathways linked with the cell cycle such as E2F_TARGETS, G2M_CHECKPOINT, MYC_TARGETS_V1, which can also be observed in the CGGA cohort (Figure 4A). Additionally, a comparison of the TCGA dataset was made to identify the pathways that differed among the C1 and C2, C1 and C3, and C2 and C3 subtypes (Figure 4B). The outcomes highlighted that the cell cycle pathway and immune-related pathways were activated in C1 patients. Therefore, we inferred that the glutamine metabolism genes used for molecular typing might exert critical effects on the cell cycle pathway and tumor microenvironment. We then used radar plots to show the pathways that were consistently and significantly activated in C1vsC2 and C2vsC3. The results showed that pathways such as G2M_CHECKPOINT and IL6_JAK_STAT3_SIGNALING were significantly activated in both the TCGA dataset and CGGA dataset (Figures 4C, D).

### Immune characteristics among molecular subtypes and their different reactions to immunotherapy/chemotherapy

**FIGURE 5:**
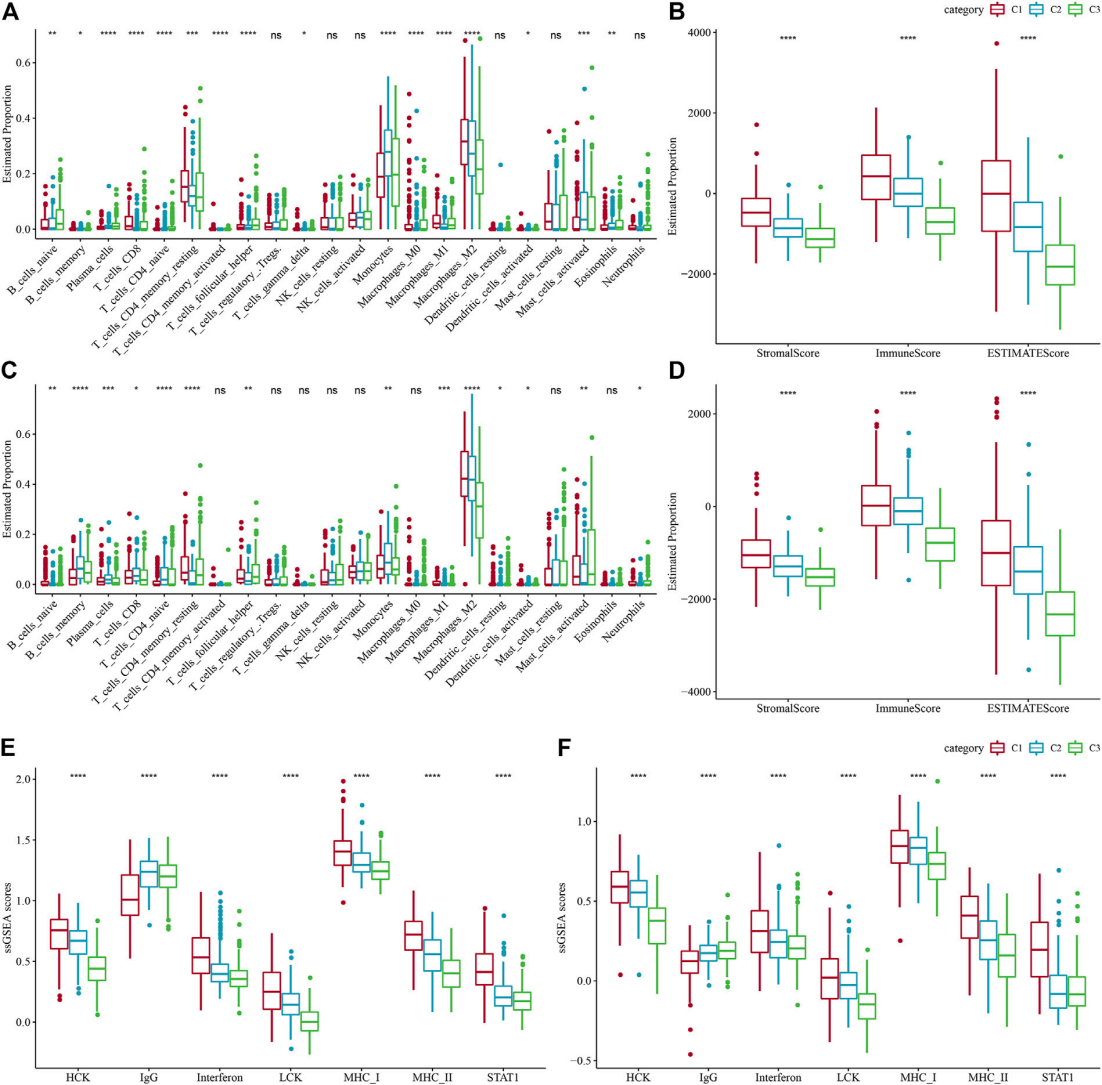
Level of immune cell infiltration in various molecular subtypes. **(A)** Differences in 22 immune cell scores between various molecular subtypes in the TCGA-LGG cohort. **(B)** Differences in 22 immune cell scores between different molecular subtypes in the CGGA cohort. **(C)** Variations in ESTIMATE immune infiltration among different molecular subtypes in the TCGA-LGG cohort. **(D)** Differences in ESTIMATE immune infiltration between different molecular subtypes in the CGGA cohort differences in ESTIMATE immune infiltration between different molecular subtypes. **(E)** TCGA-LGG cohort differences in seven inflammation-associated gene cluster scores between different molecular subtypes. **(F)** CGGA cohort variations in seven inflammation-associated gene cluster scores in various molecular subtypes.

To search deeper for the variations in the immune microenvironment of affected individuals between molecular subtypes, we assessed the immune cell infiltration level in LGG patients by targeting expression profile data using different immune cell infiltration algorithms. CIBERSORT revealed considerable variations between subtypes for almost all immune cell types, and most of the immune cell infiltration was enhanced in the C1 subtype, with Macrophages_M2 being most significantly enriched in the C1 subtype (Figure 5A). At the same time, the ESTIMATE assessment of immune cell infiltration showed that the ImmuneScore was considerably increased in the C1 subtype in comparison with the other two subtypes, indicating that C1 has a higher immune cell infiltration (Figure 5B). The same result was found in the CGGA dataset (Figures 5C, D). In addition, the inflammatory activity of the three molecular subtypes was analyzed, and the enrichment scores of seven gene sets regarding inflammation were demonstrated in the three molecular subtypes, with major variations in all six inflammatory gene sets except IgG, indicating a higher inflammatory activity in the overall C1 subtype, a phenomenon also observed in the CGGA cohort (Figures 5E, F). Low tumor purity and high enrichment of immune cells and stromal cells have been revealed to be associated with reduced overall survival in gliomas (Haddad et al., 2022). The above findings suggest that the immune infiltration level in C1 is substantially increased, and it promotes inflammation, predicting that the development of immune inflammation is likely to be responsible for the deterioration of LGG patients.

### Immune/chemotherapy treatment differences between molecular subtypes

**FIGURE 6:**
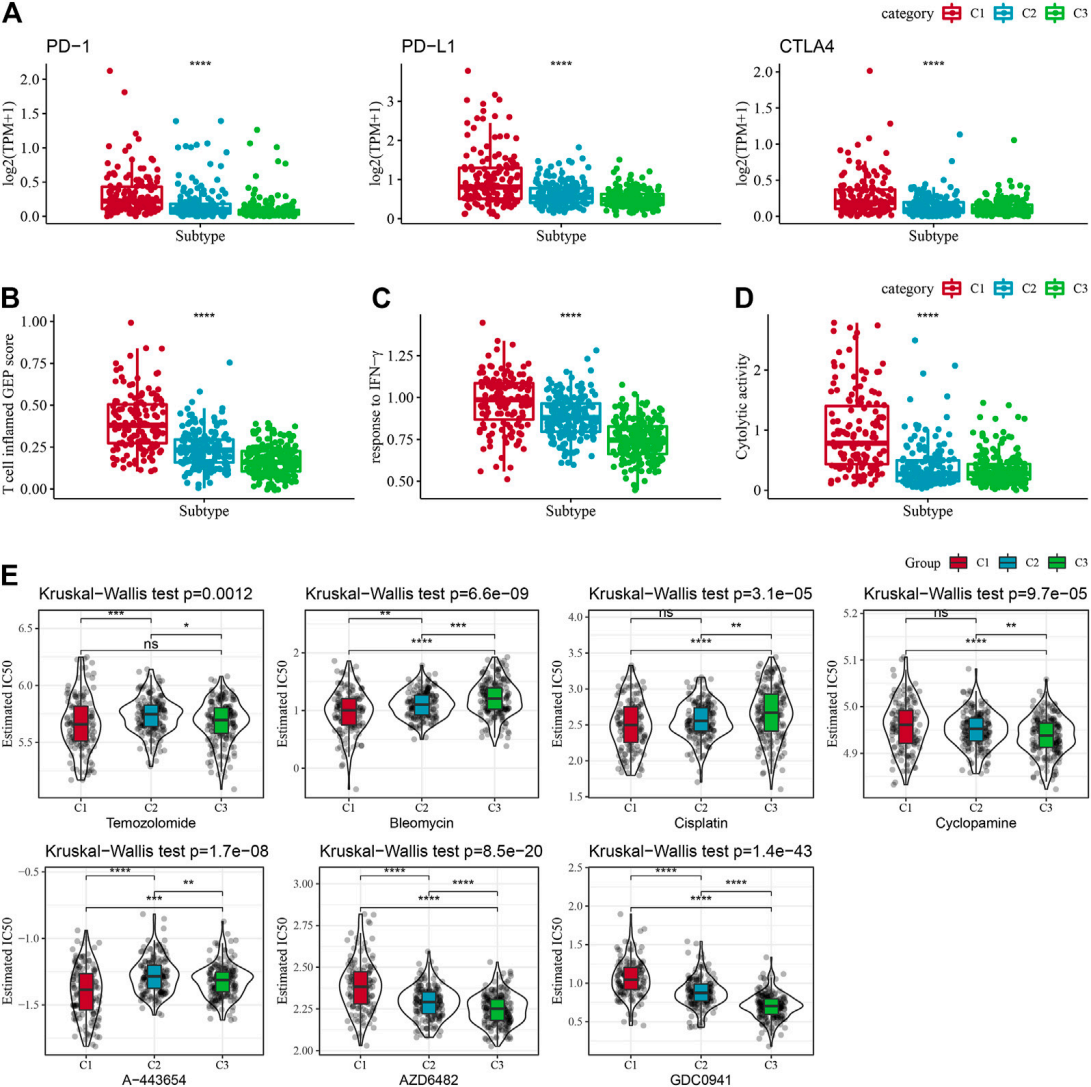
Immune characteristic scores reflecting the effect of immunotherapy for different subtypes. **(A)** Differences in T cell inflamed GEP score in molecular subtypes. **(B)** Variations in response to IFN-γ in different molecular subtypes. **(C)** Differences in expression of immune checkpoint genes between molecular subtypes. **(D)** Variations in cytolytic activity between molecular subtypes. Cytolytic activity variations. **(E)** The box plots of the estimated IC50 for Temozolomide, Bleomycin, Cisplatin, Cyclopamine, A-443654, AZD6482, GDC0941, and Bleomycin in TCGA-LGG.

Given the acknowledgment that immune checkpoint blockade (ICB) cancer immunotherapy in accordance with the inhibition of key immune checkpoints, we assessed some representative molecules and discovered that PD-1, PD-L1, and CTLA4 were significantly increasingly expressed in the C1 group (Figure 6A). On the other hand, the T-cell-inflamed gene expression profile (GEP) score was considerably enhanced in the C1 subtype (Figure 5B). In addition, we performed ssGSEA analysis for the GOBP_RESPONSE_TO_INTERFERON_GAMMA gene set and discovered that the IFN-γ response was remarkably increased in the C1 subtype (Figure 6C). In addition, we found that CYT scores, used to reflect cytotoxic effects, were remarkably higher in C1 subtypes than in other subtypes (Figure 6D). In addition, the response level of various molecular subtypes was assessed in the TCGA dataset to the conventional chemotherapeutic drugs Temozolomide, Bleomycin, Cisplatin, Cyclopamine, A-443654, AZD6482, GDC0941, and Bleomycin, and found that the C1 response to Temozolomide, Cisplatin, A-443654, and Bleomycin was more sensitive in general (Figure 6E).

### Differential expression analysis between molecular subtypes

**FIGURE 7:**
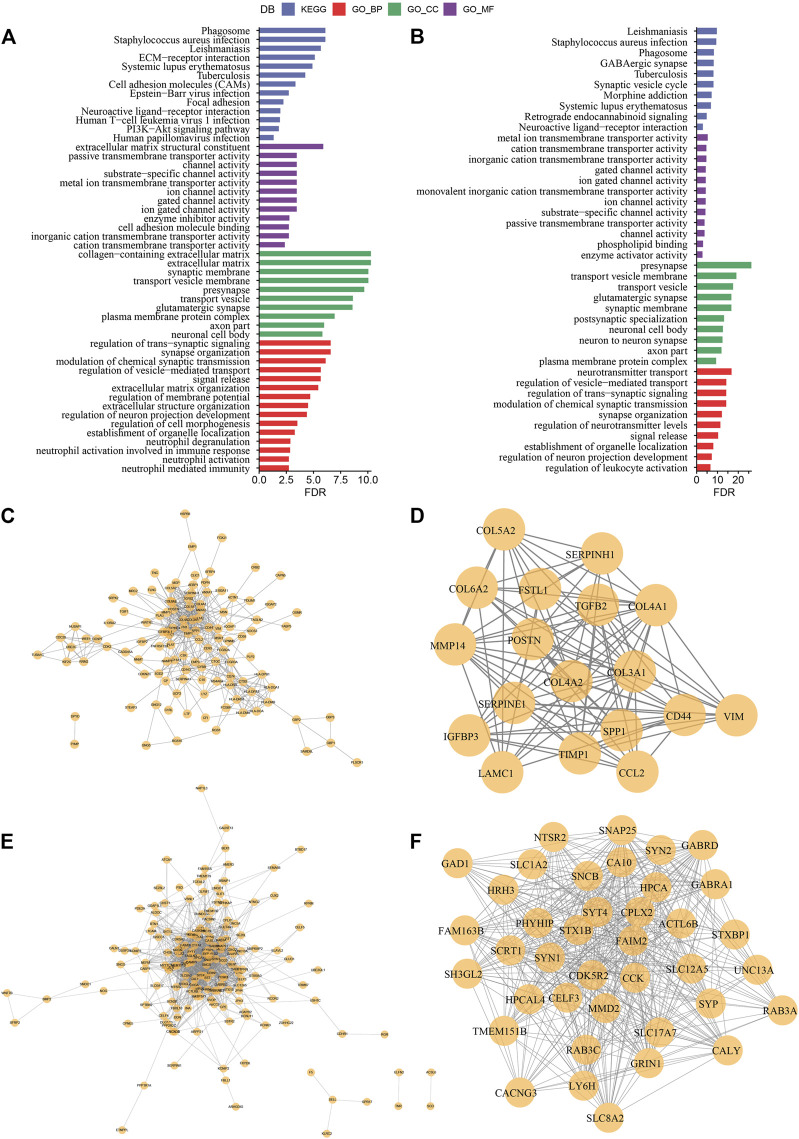
Construction of protein interaction network and key module mining **(A)** Results of GO and KEGG functional enrichment analysis of DEGs in C1 subtype; **(B)** Results of GO and KEGG functional enrichment analysis of DEGs in C3 subtype; **(C)** PPI network of differentially up-regulated genes in C1 subtype; **(D)** MCODE in PPI network of differentially up-regulated genes in C3 subtype key clusters identified by the plug-in; **(E)** PPI network of differentially down-regulated genes in C1 subtype; **(F)** Key clusters identified by the MCODE plug-in in PPI network of differentially down-regulated genes in C3 subtype.

In the previous analysis, we classified LGG samples of two independent datasets into three molecular subtypes (C1, C2, and C3). Then we identified for differentially expressed genes (DEGs) in the three different molecular subtypes by comparing C1 vs. other, C2 vs. other, and C3 vs. other. Finally, a total of 517 DEGs were discovered in C1, including 272 up-regulated genes and 245 down-regulated genes. A total of 24 DEGs were identified in C2, including eight up-regulated genes and 15 down-regulated genes. Four hundred twenty-four DEGs in total were discovered in C3, among which 260 genes were up-regulated, and 164 were down-regulated. We used the same approach to measure DEGs in different molecular subtypes in the CGGA cohort. The differential genes obtained from the two independent data sets were intersected. Further, we selected genes that differed in both data sets for functional enrichment analysis, where there were 332 co-expressed genes in C1, 317 co-expressed genes in C3 subtypes, and only ten co-expressed genes in C2 subtypes. Moreover, functional enrichment analysis of DEGs was done separately, and the enrichment outcomes of GO and KEGG pathways of genes co-expressed in C1 showed that Cell adhesion molecules, Phagosome, and Focal adhesion pathways were considerably enriched in C1. The enrichment of GO and KEGG pathways of DEGs in C3 showed that most of the pathways were not as significantly activated as in C1, which may be a key factor for the different prognostic outcomes of the two subtypes in C1 and C3 (Figures 7A, B). A PPI network was created to assess the interactions between these DEGs clearly. In this network, we used the MCODE plug-in for network module discovery and identified a total of two essential modules, and these proteins may be the key gene clusters affecting glutamine metabolism (Figures 7C–F).

### Identification of key genes for glutamine metabolism phenotype

**FIGURE 8:**
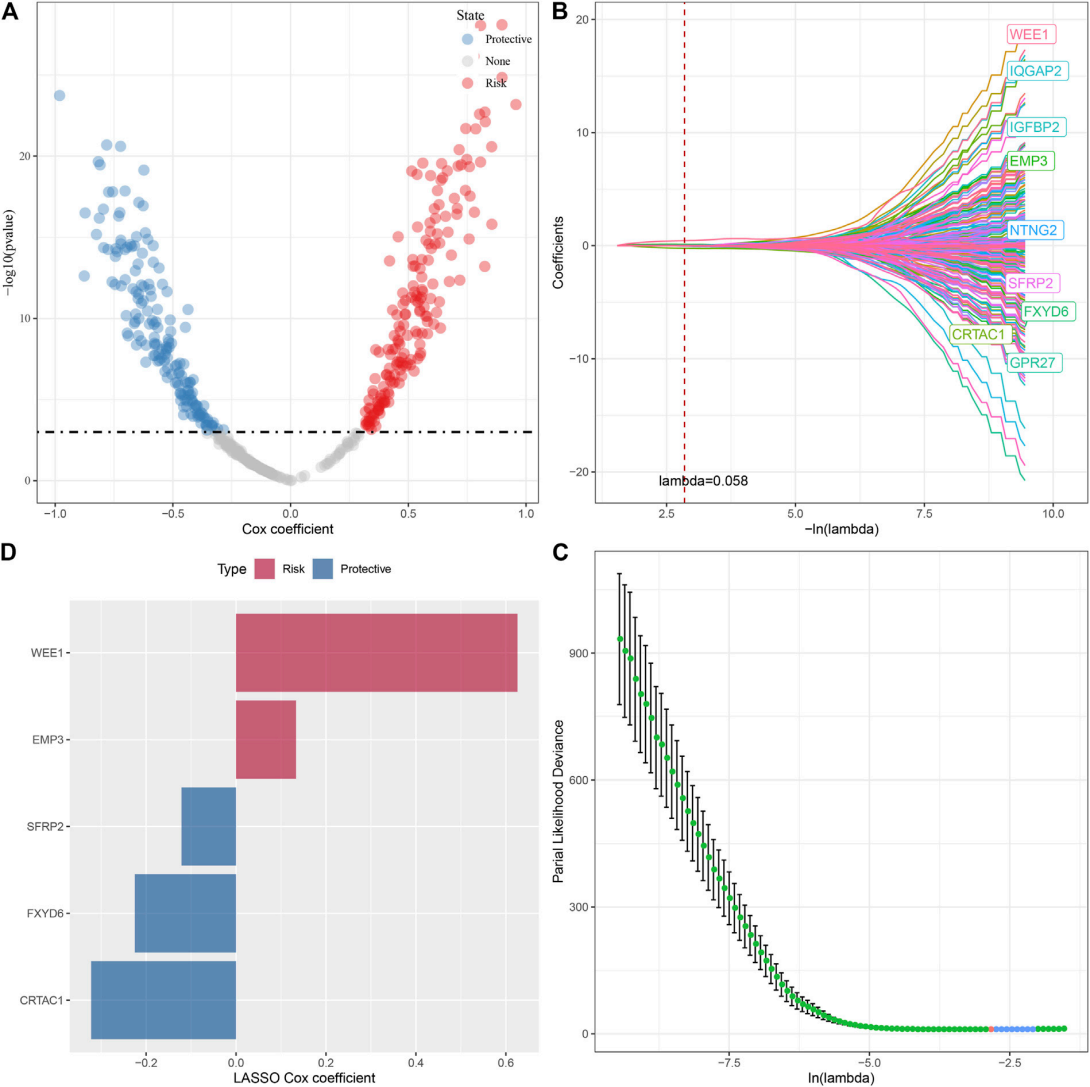
Lasso screening of key genes to construct prognostic models. **(A)** A total of 343 promising candidates were identified among the DEGs. **(B)** Trajectory of each independent variable changing along with lambda. **(C)** Confidence interval under lambda. **(D)**. Distribution of LASSO coefficients of the glutamine metabolism-related prognostic gene signature: Distribution of LASSO coefficients of the glutamine metabolism-related prognostic gene signature.

In the previous analysis, we obtained 494 DEGs after removing the duplicate genes, and next, a univariate Cox regression analysis was done to assess the DEGs; as a result, a total of 343 DEGs with high prognostic impact were identified (*p* < 0.001), including 176 Risk and 167 Protective genes (Figure 8A). Furthermore, lasso regression was utilized for further compressing these 343 DEGs to lower the genes present in the risk model. In this study, we first analyzed the independent trajectory variables individually, which highlighted that with the gradual increase in lambda, the number of independent variable coefficients tending to zero also increases gradually (Figure 8B). 10-fold cross-validation was utilized for making the model, and we analyzed the confidence intervals for each lambda, which revealed that the model was optimal at lambda = 0.058, for which we chose nine genes at lambda = 0.058 as the target genes for the subsequent step (Figure 8C). Further, based on the nine genes in the lasso analysis results, we finally identified five genes as glutamate metabolism-related genes affecting prognosis: WEE1, SFRP2, FXYD6, EMP3, and CRTAC1 (Figure 8D). The glutamate metabolism-related risk model was defined as: Risk Score = 0.626*WEE1 + 0.133*EMP3 - 0.322*CRTAC1 - 0.121*SFRP2 -0.225*FXYD6.

### Clinical prognostic modeling and validation

**FIGURE 9:**
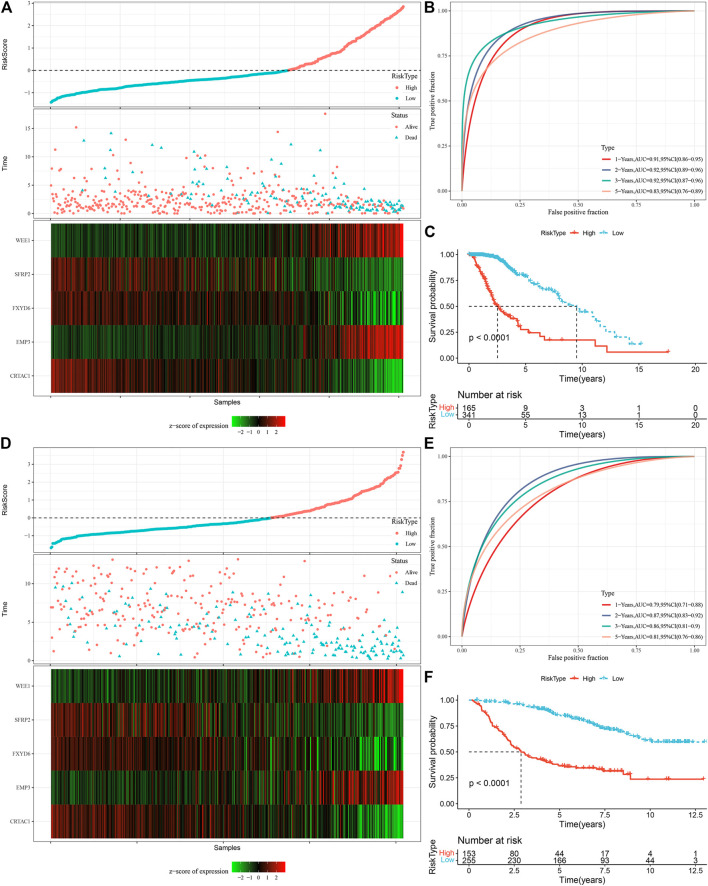
Calculation of RiskScore and determination of its robustness in two independent datasets. **(A)** RiskScore in TCGA dataset, survival time vs. survival status, and expression of the glutamine metabolism-related prognostic genes. **(B)** ROC curve of RiskScore classification in TCGA dataset. **(C)** KM survival curves of two risk groups in TCGA dataset. **(D)** RiskScore in CCGA dataset, survival time vs. survival status, and expression of the glutamine metabolism-related prognostic genes. **(E)** ROC curve of RiskScore classification in CCGA dataset. **(F)** KM survival curves of two risk groups in CGGA dataset.

We measured the individual risk scores (RiskScore) for samples, and the z-score transformation was performed according to the formula defined in our risk model. The RiskScore distribution of individuals in the TCGA training set suggested that samples having enhanced RiskScore demonstrated a worse prognosis (Figure 9A). Furthermore, we performed a ROC analysis for sorting the samples based on their RiskScore corresponding to their prognosis by means of the R software package time OC. The division of prognostic prediction efficiency was assessed at one, three, and 5 years, from which we can see that the AUC of the model is greater than 0.9, highlighting the model’s favorable reliability (Figure 9B). Finally, we classified those with RiskScore more than 0 as high-risk and those less than or equal to 0 as low-risk, in which 165 samples were sorted into a high-risk group and 341 samples into a low-risk group. Subsequently, we plotted Kaplan-Meier (KM) curves that highlighted a highly substantial variation between the prognosis of high and low RiskScore groups (Figure 9C). To confirm the robustness of the prediction of the clinical prognostic model for glutamine metabolism-related gene signatures, we performed validation in the CGGA LGG cohort, where we calculated the RiskScore of patients using the risk model of the CGGA dataset and also plotted ROC curves and survival curves, the results highlighted that the validation cohort gave us similar results as in the training set (Figures 9D–F).

### Performance of RiskScore on different clinicopathological features as well as different molecular subtypes

**FIGURE 10:**
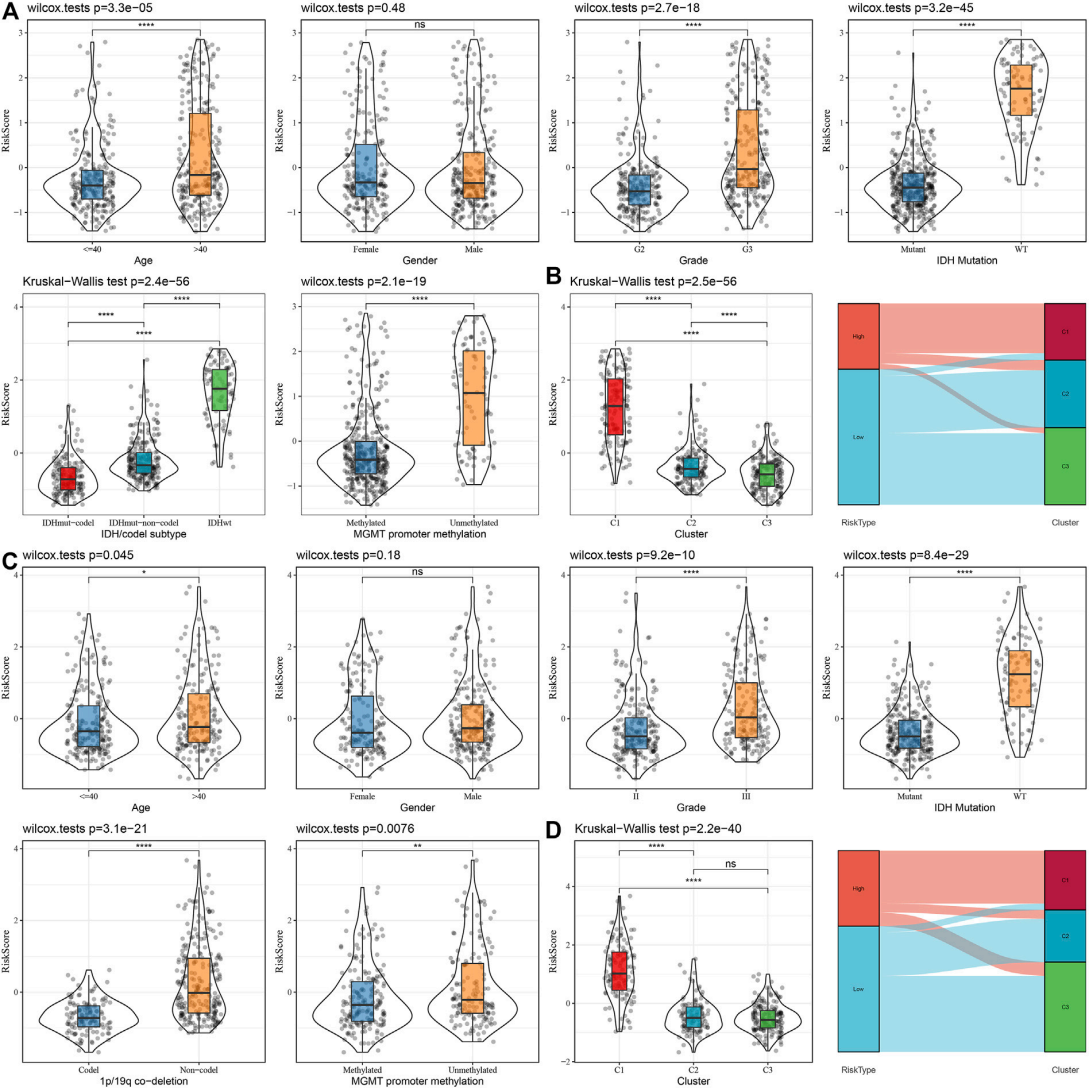
Distribution characteristics of RiskScore subgroups across clinical variables **(A)** Differences between RiskScore between different clinicopathology subgroups in the TCGA-LGG cohort. **(B)** Variations in RiskScore among different molecular subtypes and variations between molecular and RiskScore subgroups in the TCGA-LUAD cohort. **(C)** Differences between RiskScore between different clinicopathological subgroups in the CGGA cohort. **(D)** Differences between RiskScore between different molecular subtypes and differences between molecular subtypes and RiskScore subgroups in the CGGA cohort.

To assess the correlation of RiskScore with the clinical properties of LGGs, we analyzed the variations in the RiskScore scores in different TNM grades and stage clinical grades in the TCGA dataset. The outcomes highlighted that the RiskScore score enhanced with the increase in clinical grade. Therefore, samples with increased clinical grades had increased RiskScore scores. Patients aged above 40 possessed worse prognostic outcomes. IDH wild-type and MGMT hypermethylation also demonstrated their riskiness (Figure 10A). Moreover, we compared the differences in RiskScore across molecular subtypes and found that C1 had the worst prognostic outcome while also having the highest RiskScore, and the Sankey diagram also demonstrated a higher proportion of patients with RiskScore-High in C1 (Figure 10B). We also replicated the results in the CGGA dataset (Figures 10C, D).

### Immune infiltration/pathway characteristics between RiskScore subgroups

**FIGURE 11:**
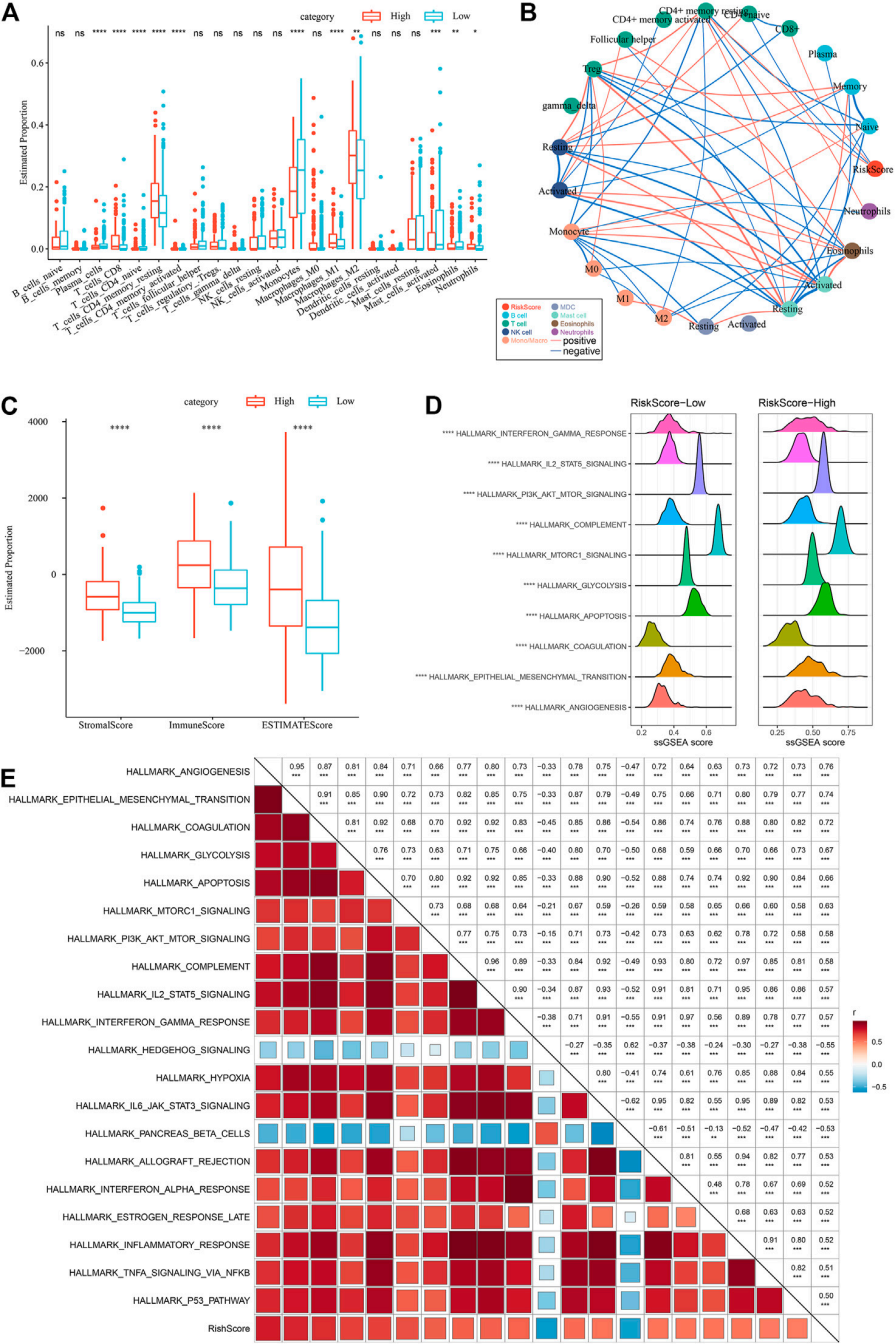
Different immune cells with different infiltration levels in the RiskScore grouping. **(A)** Proportion of immune cell components in the TCGA cohort. **(B)** Correlation analysis between 22 immune cell components and RiskScore in the TCGA cohort. **(C)** Proportion of immune cell components calculated by ESTIMATE software in the TCGA cohort. **(D)** Top10 pathways with the most major variations between RiskScore-High and RiskScore-Low. **(E)** Results of correlation analysis between KEGG pathways with RiskScore correlations greater than 0.5 and RiskScore.

To highlight the variations in the immune microenvironment of individuals in the RiskScore subgroups, the relative abundance of 22 immune cell types was compared in the high and low RiskScore subgroups by expression profiling in the TCGA dataset, and it could be observed that some of the immune cells were substantially varied in the high and low RiskScore subgroups (Figure 11A). Additionally, we assessed the link of RiskScore with 22 immune cell components and could see that RiskScore showed a positive correlation with most immune cells, such as M2 phase macrophages (Figure 11B). In addition, ESTIMATE was employed to analyze immune cell infiltration, and it was observed that ImmuneScore was remarkably increased in the RiskScore-High group in comparison with the RiskScore-Low group, with higher immune cell infiltration (Figure 11C). These results are identical to those of C1, which has higher immune infiltration and a worse prognostic outcome than other subtypes. Furthermore, the pathways for the variations between RiskScore-high and RiskScore-low groups were compared, and it was observed that RiskScore-high was significantly enriched in some cancer-related pathways such as HALLMARK_ GLYCOLYSIS, HALLMARK_PI3K_AKT_MTOR_SIGNALING, HALLMARK_EPITHELIAL_MESENCHYMAL_TRANSITION, *etc.* (Figure 11D).

Therefore, the correlation between the enrichment scores of these pathways and the RiskScore was measured, and the pathways with a correlation greater than 0.5 were chosen, as illustrated in Figure 11E, from which it can be seen that the RiskScore showed a positive correlation with cancer-related pathways such as HALLMARK_HYPOXIA, HALLMARK_GLYCOLYSIS, HALLMARK_ EPITHELIAL_MESENCHYMAL_TRANSITION, *etc.* (Figure 11E).

### Differences in reaction to immunotherapy/chemotherapy among RiskScore subgroups

**FIGURE 12:**
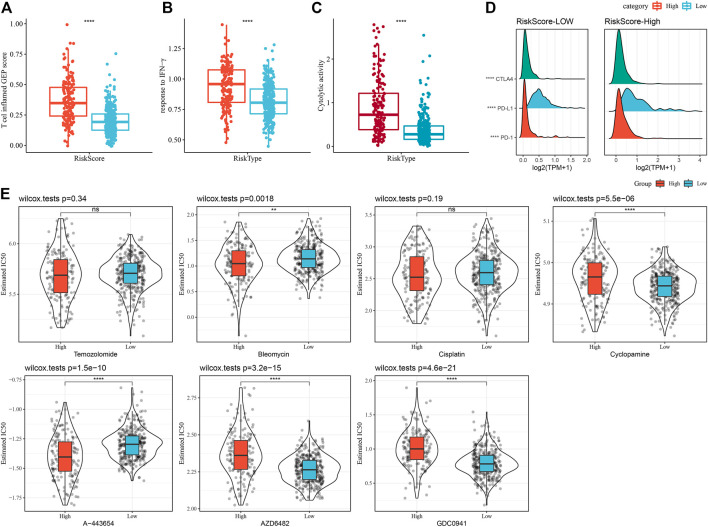
Immune characteristic scores reflect the effect of immunotherapy in different RiskScore subgroups. **(A)** Differences in T cell inflamed GEP scores between different molecular subtypes. **(B)** Variations in response to IFN-γ between different molecular subtypes. **(C)** Variations in cytolytic activity between different molecular subtypes. **(D)** Differences in expression of immune checkpoint genes in various molecular subtypes. **(E)** The box plots of the estimated IC50 for Temozolomide, Bleomycin, Cisplatin, Cyclopamine, A- 443654, AZD6482, and GDC0941 in TCGA-LGG.

A series of immune signature scores were employed to assess the immunotherapy response in the RiskScore subgroups. The T-cell-inflamed GEP score was considerably enhanced in the RiskScore-High group (Figure 12A). The IFN-γ response was elevated considerably in the RiskScore-High subgroup (Figure 12B). In addition, we found that CYT scores, which are used to reflect cytotoxic effects, were substantially increased in the RiskScore-High group than in other subtypes (Figure 12C). Considering that ICB cancer immunotherapy works by inhibiting key immune checkpoints, we assessed certain representative molecules and discovered that PD-1, PD-L1, and CTLA4 were significantly more increasingly expressed in the high RiskScore group (Figure 12D). The response of various molecular subtypes was assessed in the TCGA dataset to the traditional chemotherapeutic agents, Temozolomide, Bleomycin, Cisplatin, Cyclopamine, A-443654, AZD6482, GDC0941, and Bleomycin and found that overall RiskScore-High was more sensitive to A-443654 and Bleomycin (Figure 12E).

### RiskScore combined with clinicopathological properties for further improvement of prognostic models and survival prediction

**FIGURE 13:**
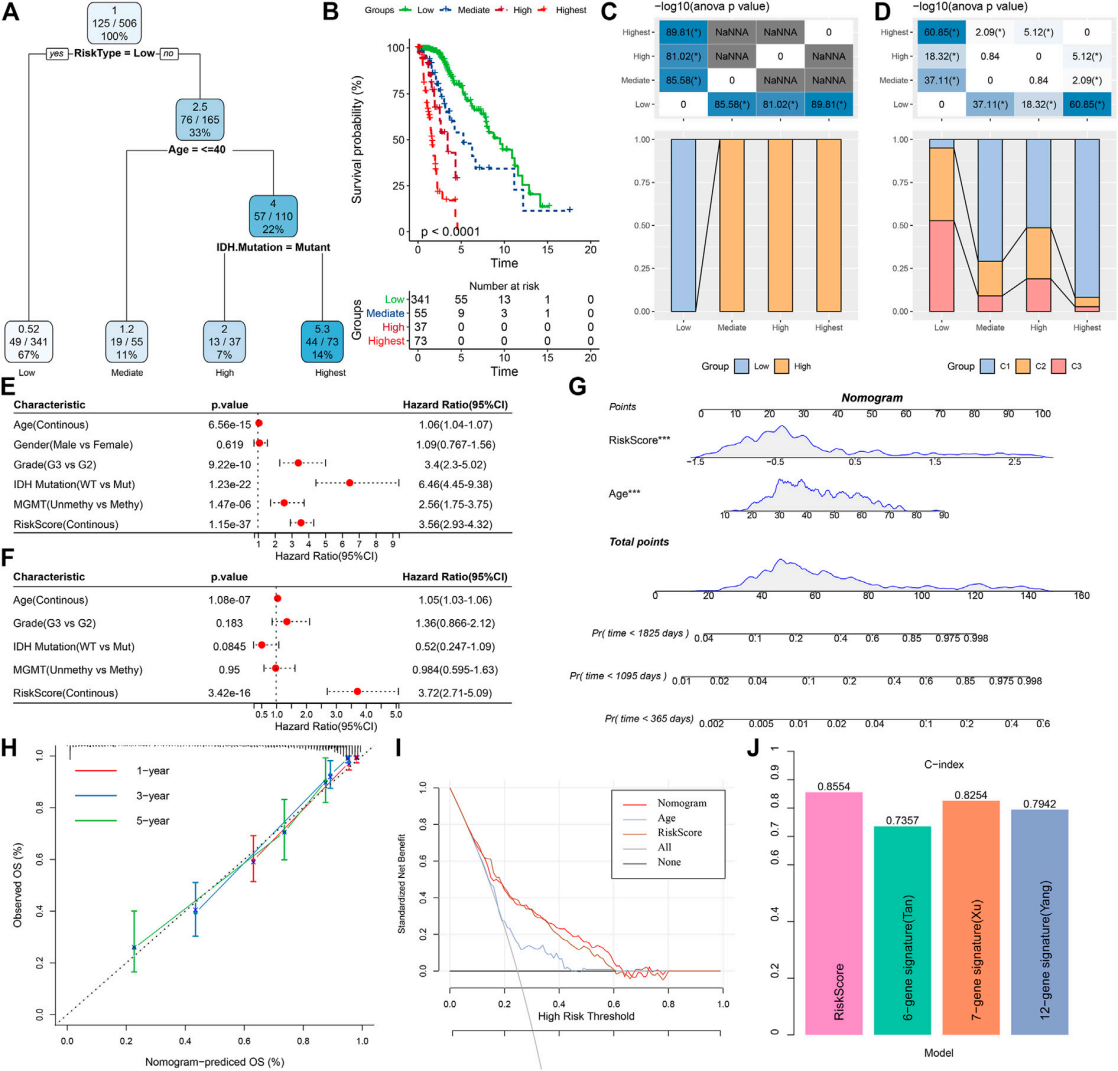
Determining optimal prognostic factors and determining their reliability by decision tree **(A)** Individuals with full-scale annotations including RiskScore, age, gender, and TNM stage were employed to develop a survival decision tree for optimizing risk stratification. **(B)** Major variations of overall survival were observed among the four risk subgroups. **(C,D)** Comparative analysis among different subgroups. **(E,F)** Univariate and multivariate Cox analysis of RiskScore and clinicopathological properties. **(G)** Columnar line plot model. **(H)** Calibration curves for 1, 3, and 5 years for columnar line plots. **(I)** Decision curves for columnar line plots. **(J)** C-index of our risk model and other previously reported risk models.

In this research, a decision tree was built according to the patient’s age, gender, TNM Stage pathology information, and RiskScore in the TCGA dataset, which showed that only RiskType, Age, and IDH Mutation were a part of the decision tree identifying four distinct risk subgroups; and RiskType was the most powerful parameter among them (Figure 13A). There was a significant difference in overall survival among the four risk subgroups (Figure 13B). Among the risk subgroups, including Mediate, High, and Highest, all patients were shown to be RiskScore-High. Moreover, variations in the distribution of our defined molecular subtypes were found in the various risk subgroups, with the Highest risk subgroup being more occupied by our defined molecular subtype C1 subtype (Figures 13C, D). Univariate and multivariate Cox regression analysis of RiskScore and clinicopathological properties revealed RiskScore as the most significant prognostic factor. The HRs of 3.56 and 3.72 in the two datasets were significantly greater than 1, respectively, predicting that RiskScore is a risk factor for individuals with LGG (Figures 13E, F). For risk assessment quantification and survival probability of individuals with LGG, RiskScores and other clinicopathological features were taken collectively to create a column line plot, and the model outcomes highlighted that RiskScore had the most significant impact on survival prediction (Figure 13G). We further analyzed the model’s prediction accuracy using the calibration curve and observed that the prediction calibration curves at the three calibration points of 1, 3, and 5 years nearly overlapped with the standard curve, which suggested that the column line plot had favorable predictive performance (Figure 13H). The model’s reliability was also assessed using DCA, and the outcomes highlighted that both RiskScore and Nomogram benefits were significantly higher than the extreme curves, and both nomogram and RiskScore showed the strongest survival prediction ability compared to other clinicopathological features (Figure 13I). In addition, our risk model also showed higher C-index compared with other models in the previous studies (He et al., 2020; Tang et al., 2020; Wu et al., 2021) (Figure 13J).

## Discussion

In this research, we explored the prognostic significance of glutamine metabolism genes in LGG using univariate COX regression in two independent datasets and furthermore selected genes that were significant in both datasets, with glutamate--cysteine ligase regulatory subunit (GCLM) having the highest risk ratio and glutamate dehydrogenase 1 (GLUD1) having the lowest risk ratio. Although in LGG, these two genes have not been reported frequently, other studies have reported them coding for enzyme classes associated with glutamine metabolism. GCLM is the first rate-limiting enzyme of glutathione synthesis with an amino-cysteine ligase activity (Diaz-Hernandez et al., 2005). GLUD1 acts as glutamate dehydrogenase, catalyzing the oxidative deamination of glutamate to *α*-ketoglutarate and ammonia (Fang et al., 2002). For the 17 prognostically significant glutamine metabolism genes, the data set was divided into three subtypes, of which the C1 subtype had the worst prognosis and the lowest glutamine metabolism enrichment score. Among the different clinical variables, the predominance of C1 patients was older than 40, indicating that increasing age is also a risk factor for patient prognosis. In contrast, among the gender variables, there was no difference between the three subtypes, suggesting that gender does not affect the prognosis of LGG patients. Patients with MGMT hypermethylation were more predominant in the C1 subtype, and in combination with previous work, we know that MGMT methylation levels are significantly associated with patient prognosis. The IDH mutation patient group had a prolonged overall survival (OS) of 9.4 years and the OS being 5.7 years for patients receiving radiotherapy alone, while in the wild-type IDH patient group, the median survival of patients on the radiotherapy alone regimen was 1.8 years (Cairncross et al., 2014); this is in line with our best prognosis for the C3 subtype. Moreover, in C1, LGG patients had a higher frequency of mutations, with significantly higher scores for TMB, Aneuploidy Score, and Homologous Recombination Defects than for other subtypes. In C1, quite a few pathways are linked with cell cycle and metastatic invasions, such as E2F_TARGETS, G2M_CHECKPOINT, MYC_TARGETS_V1, and EPITHELIAL_MESENCHYMAL_TRANSITION. This indicates that in C1, the patients are more malignant and more prone to invasion. These indicate a possible association of glutamine with these differentially activated pathways. It is known from previous reports in the literature that inhibition of ASCT2 in prostate cancer is accompanied by decreased glutamine uptake, which significantly inhibits tumor growth and metastasis *in vivo* through the cell cycle progression of E2F transcription factors (Wang et al., 2015). The literature reports that cell-intrinsic programs can drive preferential access to glucose and glutamine for immune and cancer cells, respectively, providing substantial energy for the tumor microenvironment and cancer cell proliferation development (Reinfeld et al., 2021).

To explore the tumor microenvironment of C1, we assessed the infiltration level of different immune cells in different subtypes; both ImmuneScores and matrix scores were found to be significantly elevated in C1, most notably in M2 stage macrophages, indicating that these particular immune cells are prominently involved in promoting the progression of LGG progression. Immune checkpoints such as PD-1 and PD-L1 were significantly highly expressed in C1 subtypes, while T-cell-inflamed GEP score and response to IFN-γ response were significantly elevated in C1 subtype, suggesting that ICB-based cancer immunotherapy regimens may be effective in LGG patients. In addition, we found that C1 was more sensitive to Temozolomide, Cisplatin, A-443654, and Bleomycin, but not to several other conventional drugs, suggesting that physicians could improve their regimens when using conventional drugs for LGG.

Following differential analysis of different subtypes and functional enrichment analysis of common differential genes, we found that most of the pathway activation, such as Cell adhesion molecules and Focal adhesion, were not as significant as C1, which may be one of the key factors for the different prognostic outcomes of the two subtypes C1 and C3. Based on the protein interaction data, two gene expression patterns with different prognostic outcomes could be seen. We constructed the prognostic model and then determined its robustness and reliability by ROC and calculated the RiskScore for each patient. By examining the distribution of RiskScore among different clinical variables, it can be found that RiskScore is strongly associated with age, Grade, IDH mutation status, and MGMT methylation level. Most of the immune cell infiltration degrees and cancer-related pathways also have a significant correlation with RiskScore. Moreover, patients in the RiskScore-High group were more responsive to two traditional drugs, A-443654 and Bleomycin.

Finally, we determined the most significant prognostic factors by constructing a decision tree, which showed that patients with RiskType of HIGH, age over 40, and no mutation in IDH had the worst prognosis. Afterward, we found that RiskScore had the greatest effect on survival prediction by column line plot and plotted the calibration curve and DCA to ensure the reliability and accuracy of the model.

## Conclusion

Initially, we used glutamine metabolism-related genes to identify stable molecular subtes by consistent clustering, and these three molecular subtypes have different prognostic, pathological, pathway, and immunological characteristics. Afterward, we screened a total of five key genes related to glutamine metabolism phenotype by DEGs between molecular subtypes and lasso, then we constructed a clinical prognostic model based on the key genes associated with glutamine metabolism phenotypes which was robust and independent of clinicopathological features and showed stable predictive efficacy in independent datasets.

Finally, we combined RiskScore with clinicopathological features using a decision tree model to improve the prognostic model and survival prediction ability.

## Data Availability

The original contributions presented in the study are included in the article/Supplementary Material, further inquiries can be directed to the corresponding author.
